# Plasmon-assisted site-selective growth of Ag nanotriangles and Ag-Cu_**2**_O hybrids

**DOI:** 10.1038/srep44806

**Published:** 2017-03-21

**Authors:** Ying Xie, Liang Ma, Zi-Qiang Cheng, Da-Jie Yang, Li Zhou, Zhong-Hua Hao, Qu-Quan Wang

**Affiliations:** 1 Key Laboratory of Artificial Micro- and Nano-structures of the Ministry of Education and School of Physics and Technology, Wuhan University, Wuhan 430072, P. R. China; 2 The Institute for Advanced Studies, Wuhan University, Wuhan 430072, P. R. China

## Abstract

We report a plasmon-assisted growth of metal and semiconductor onto the tips of Ag nanotriangles (AgNTs) under light irradiation. The site-selective growth of Ag onto AgNTs are firstly demonstrated on the copper grids and amine-coated glass slides. As the irradiation time increases, microscopic images indicate that AgNTs gradually touch with each other and finally “weld” tip-to-tip together into the branched chains. Meanwhile, the redshift of plasmon band is observed in the extinction spectra, which agrees well the growth at the tips of AgNTs and the decrease of the gaps between the adjacent nanotriangles. We also synthesize AgNT-Cu_2_O nanocomposites by using a photochemical method and find that the Cu_2_O nanoparticles preferably grow on the tips of AgNTs. The site-selective growth of Ag and Cu_2_O is interpreted by the local field concentration at the tips of AgNTs induced by surface plasmon resonance under light excitation.

Different metal nanocrystals with various morphologies, such as nanospheres, nanoplates, nanorods, have been used in many fields, such as surface-enhanced Raman scattering (SERS), optical sensing, near-field optical probing, and optical bio-imaging^1^,^2^,^3^,^4^,^5^,^6^,^7^,^8^. Among all, the morphologies are controlled by the overgrowth of nanoparticles which are reduced by metal precursors. Especially, the overgrowth on nanoparticles can be used for site-selective deposition of metal atoms on specific facets^9^,^10^,^11^,^12^. When the adjacent metal nanoparticles approach with each other, plasmon coupling between adjacent nanoparticles can produce a new hybridized resonance band, and local electromagnetic field at the nanogap will be largely enhanced^13^,^14^,^15^. With these excellent characters, the assembled metal nanostructures have also been used for higher SERS signals^16^,^17^,^18^,^19^,^20^,^21^,^22^,^23^,^24^,^25^.

Many approaches have been used to control the assembly of metal nanostructures, especially gold and silver nanostructures. The typical assembly methods include solvent-induced evaporation^26^,^27^,^28^,^29^, biological templating method^30^,^31^,^32^,^33^, polymer grafted method^34^,^35^,^36^,^37^, electrostatic interaction-directed method^38^,^39^,^40^ and e-beam lithography^41^. For example, Xu *et al*. have found that the monodisperse Au polyhedrons could be assembled into two-dimensional nanostructures with the assistance of amphiphilic ligands^26^. Ag nanocubes and AgNTs grafted with polymer chains of varying lengths are assembled into oriented dimers or one-dimensional strings^34^. Using the polymer-directed assembly, Tao and his co-workers have reported that the assembled AgNTs could be used as bowtie antennas with tunable optical properties^36^. Electron-beam lithography can also be regarded as an approach to manufacture assembled nanostructures with fixed gap about 10–15 nm. However, these fabricating processes for the assembly of nanostructures are complicated or require surface chemical modification.

Self-assembled nanostructures can also be controlled by photochemical method. Liz-Marzán and his co-workers have reported tip-to-tip welding of gold nanorods can be form with excitation by laser irradiation^42^. Yoon and his co-workers have reported that two adjacent Au nanoparticles could gradually approach to each other with continuously tunable nanogap under UV irradiation^43^. Compared with the conventional fabrication techniques, the photochemical method can not only produce site-selective assembly, but also tune the gap distance continuously. The photochemical method can also be applied to synthesize metal-semiconductor hybrids. Especially, Ag-Cu_2_O nanocomposites are widely investigated due to their excellent photocatalytic activities over an extend wavelength range. Core-shell Ag-Cu_2_O has been synthesized in wet-chemical method^44^,^45^. However, Ag-Cu_2_O nanocomposites synthesized by using photochemical method have seldom been reported.

In this paper, AgNTs are prepared by a photochemical method reported by Chad Mirkin^46^. Most importantly, we demonstrate a site-selective growth of Ag onto the AgNTs assisted by surface plasmon resonance (SPR). The result is firstly demonstrated on copper grids and amine-coated glass slides. We have shown that the plasmon-assisted site-selective growth of Ag leads to the tip-to-tip assembly of AgNTs in a large region under light irradiation. As the irradiation time increases, a wide range of AgNTs touch with each other at the tips and finally assemble together on the substrates due to the concentrated local field induced by surface plasmon resonance. A redshift of SPR peak wavelength is observed as the irradiation time increases. For the purpose of further researching the site-selective growth mechanism induced by light irradiation, we have synthesized the Ag-Cu_2_O hetero-nanostructures by a photochemical process and site-selective growth of Cu_2_O on the AgNTs are observed.

## Results and Discussion

### Growth of AgNTs

**Figure 1:**
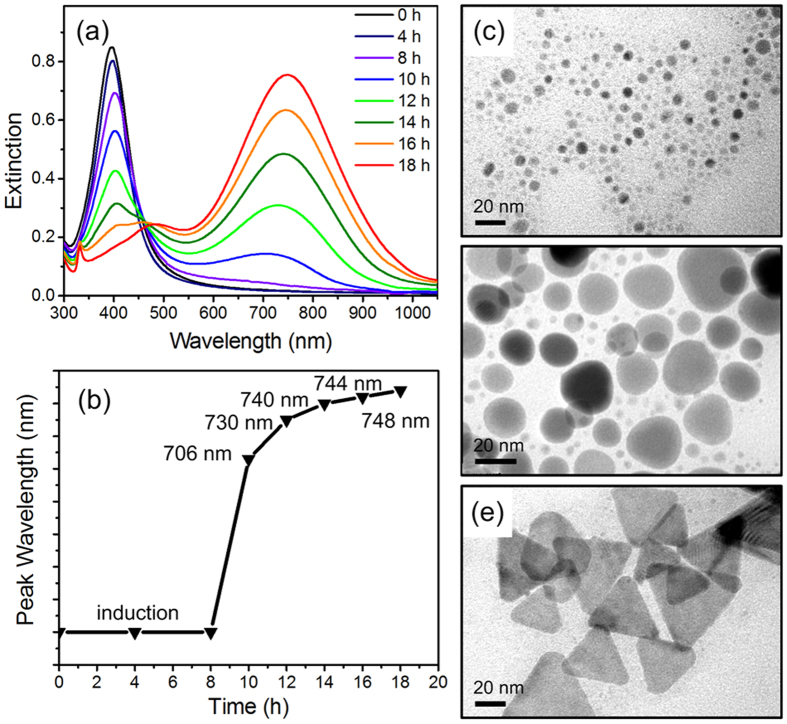
(**a**) Extinction spectra of the silver seed solutions with different reaction time of light irradiation. (**b**) Corresponding plasmon peak wavelength varying with reaction time. TEM images showing the morphology at the reaction time of (**c**) 0 h, (**d**) 10 h, and (**e**) 18 h.

The extinction spectra of the silver seeds solution were measured at selected reaction time, as plotted in Fig. 1a. Peak wavelength changes as the reaction time increases (Fig. 1b). The SPR wavelength of the original Ag seeds locates at ~398 nm (Fig. 1a, black curve). With the reaction time increasing, the intensity of plasmon peak in UV region gradually decreases and the in-plane dipole plasmon peak located at infrared region appears. After irradiation for 18 h, the in-plane dipole plasmon wavelength shows an evident red-shift to 748 nm (Fig. 1a, red curve). The extinction spectra of reaction solutions without light irradiation were also measured at 30 °C (Figure S2). The peak wavelength is almost unchanged as the reaction time increases. It indicates the light irradiation drives the growth of Ag triangle shape due to the reduction of Ag ions.

TEM analysis confirms the formation process of AgNTs in solution. Figure 1c shows the Ag seeds before irradiation. After 10 h of reaction time, Ag nanoplates are produced as shown in Fig. 1d. After 16 h of reaction time, nearly all of the Ag nanoplates are converted to the triangular shape (Fig. 1e). The average edge length of AgNTs are measured about ~50 nm.

### Plasmon-assisted site-selective growth and assembly of AgNTs on substrates

**Figure 2:**
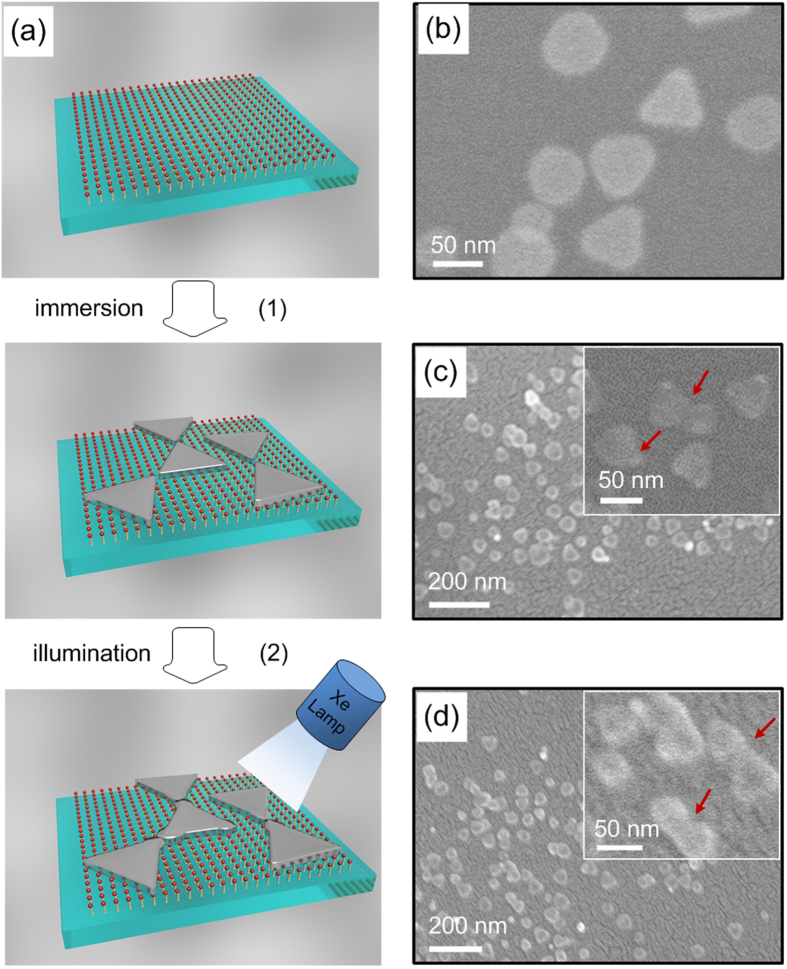
(**a**) Schematic illustrations of stepwise procedure of AgNTs assembly on glass slides using a photoinduced method. SEM images showing the morphology of AgNTs before irradiation (**b**), after irradiation for 2 h (**c**), and after irradiation for 4 h (**d**).

The processes of site-selective growth and assembly of AgNTs on glass slides by using a photochemical method are illustrated in Fig. 2a. The glass slide coated with amine groups produces a positively charged surface after immersion in an ethanol solution of APTMS^37^,^38^. PVP-coated AgNTs is negatively charged. The amine-coated glass slides are put into the AgNTs solution. The AgNTs are adsorbed on the amine layer by electrostatic interactions. Then, the glass slides with AgNTs are illuminated with a Xe lamp.

For monitoring the plasmon band evolution, we choose transparent glass slides to adsorb the AgNTs. The AgNTs on amine-coated glass slides form monolayer to avoid the aggregation of nanoplates. The morphological changes of AgNTs on glass slides with different light irradiation time are investigated by SEM. Before irradiation, the AgNTs adsorbed on the amine-coated glass slides show perfect edge (Fig. 2b). As the irradiation time increases, the shapes of AgNTs are varied with the growth of Ag on the tips (Fig. 2c). With the increased illumination time, the tip-to-tip assembled and the “welded” AgNTs are observed in Fig. 2d.

**Figure 3:**
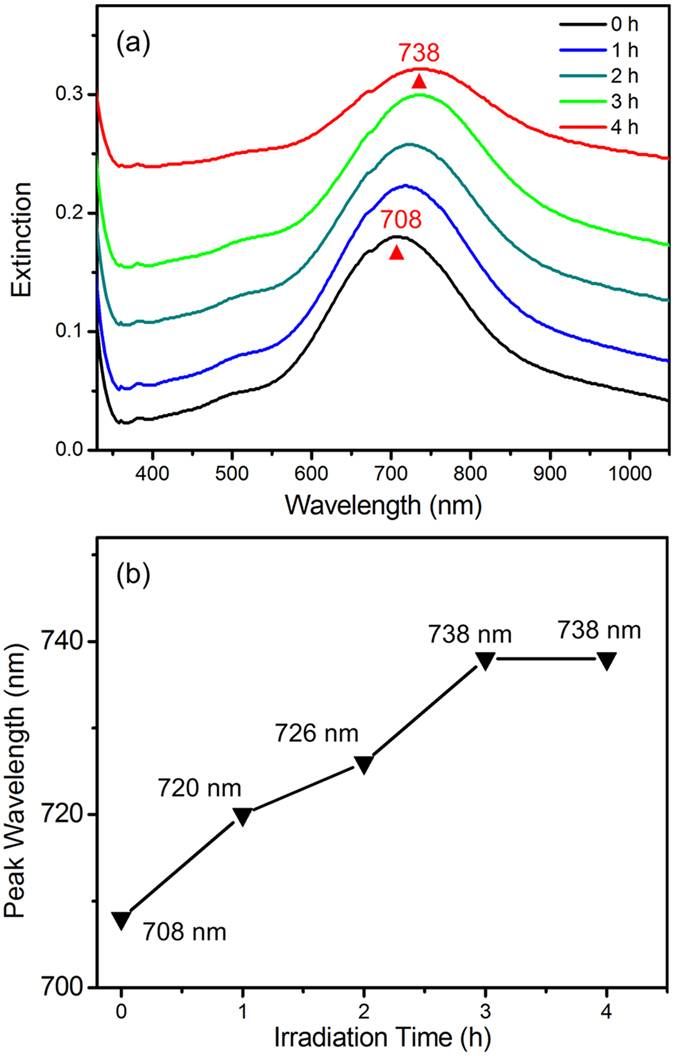
(**a**) Extinction spectra of AgNTs on glass slides with different times of light irradiation. (**b**) Plasmon peak wavelengths as a function of light irradiation time.

As shown in Fig. 3a, the extinction spectra changes as the irradiation time increases. The plasmon band evolution is monitored to understand the plasmon-assisted growth. The peak wavelength as a selected irradiation time is plotted in Fig. 3b. The black solid triangle at 0 h represents the extinction spectrum of the original AgNTs adsorbed on glass slides with a SPR peak at ~708 nm. As the irradiation time increases, the plasmon coupling band slowly redshifts to 738 nm and experiences a slightly width broadening. We have compared the plasmon band variation of samples on substrate with and without light irradiation. As shown in Figure S3, the plasmon band redshifts 30 nm with light irradiation. The sample covered by a tin foil paper only shows 9 nm shift. The results imply that the light irradiation is critically important for the SPR redshift as well as the site-selective growth of Ag.

**Figure 4:**
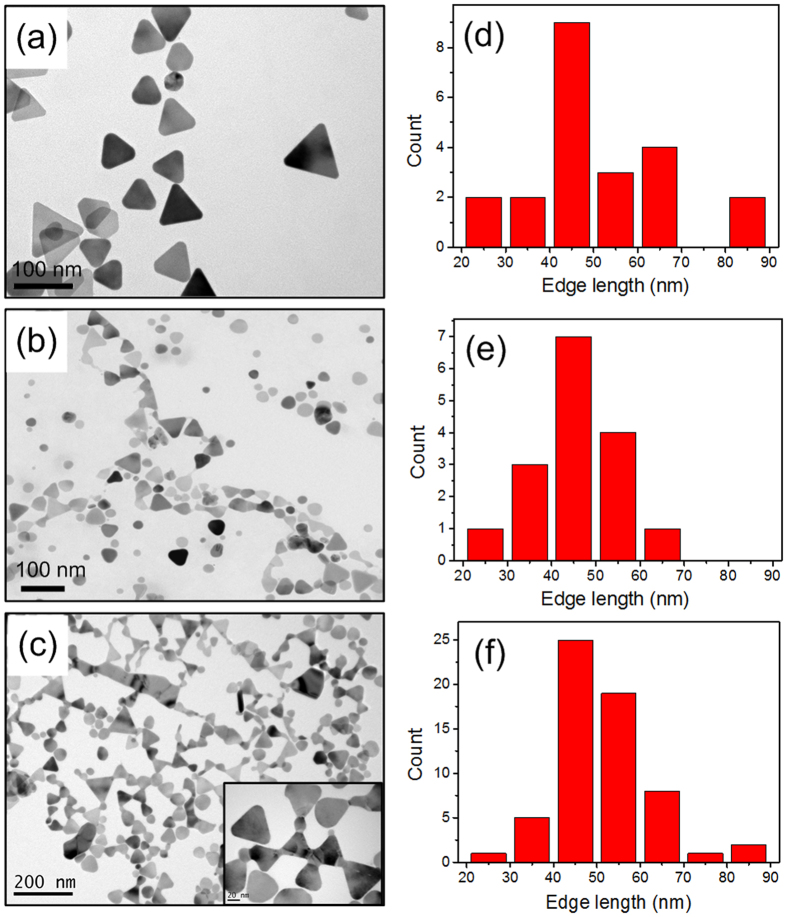
TEM images of AgNTs morphology evolution on copper grids. (**a**) Before irradiation. (**b**) After irradiation for 2 h. (**c**) After irradiation for 5 h (Inset: high magnification TEM image of the assembled AgNTs). The corresponding histograms of triangle dimension are shown in (**d**–**f**).

The as-prepared AgNTs are dropped on copper grids and then irradiated by the Xe lamp. Figure 4a shows the original AgNTs on the copper grid without irradiation. Morphological changes are observed by TEM as the irradiation time increases. After irradiation for a while, the TEM image exhibits that AgNTs have grown a new tip, and many adjacent AgNTs touch each other due to the growth of Ag on the tips (Fig. 4b). Extending the irradiation time, the adjacent AgNTs are “welded” together and assembled into large-scale branched chains. A wide range of AgNTs are finally “welded” together (Fig. 4c). The corresponding histograms of triangles dimensions are shown in Fig. 4d–f. The average edge length of AgNTs is almost unchanged before and after Ag growth irradiated by light. The results indicate that Ag atoms are almost deposited on the tips rather than on the side edges of the AgNTs under the light irradiation.

The site-selective growth of Ag and the assembly of AgNTs on the substrates is originated from the plasmon-assisted growth. Light irradiation induces the free conduction electrons in metal, called surface plasmons. The SPRs lead to the strongly concentrated local field at the tip regions of AgNTs. In reaction solution for AgNTs growth, light irradiation drives the growth of Ag triangle shape due to overgrowth of Ag reduced by Ag precursors. When AgNTs in growth solution are dropped onto the substrate, the light irradiation would also manipulate the morphology variation and the plasmonic responses of Ag^47^,^48^,^49^,^50^,^51^,^52^. Largely enhanced local field around the tips could accelerate the light-driven reduction of Ag near the tip regions of AgNTs, which results in the migration of silver atoms to the tips and the subsequent site-selective growth^53^,^54^,^55^,^56^. The relaxation of plasmon also produces the local thermal effect which may help the localized chemical reaction of Ag reduction. Furthermore, the sharp corner of AgNTs could be considered as the nucleation for the growth of silver atoms, due to high activity at the tips^57^. As shown in Fig. 4b,c, the microscopic observations exhibit that AgNTs have grown new tips and then are “welded” together.

### Plasmon-assisted site-selective growth of Ag-Cu_2_O hetero-nanostructures

**Figure 5:**
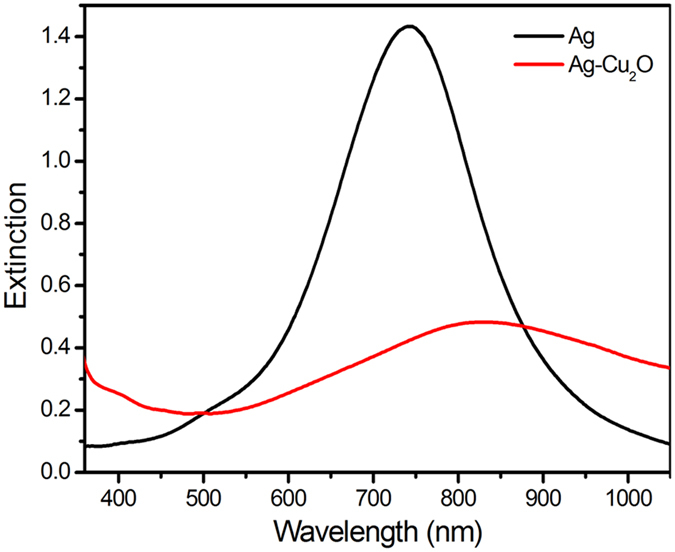
Extinction spectra of AgNTs and Ag-Cu_2_O hetero-nanostructures.

**Figure 6:**
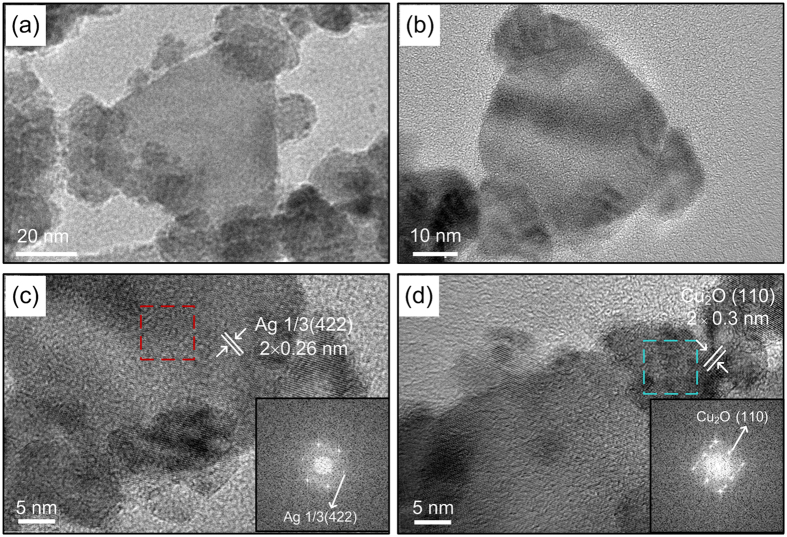
(**a**,**b**) TEM images of Ag-Cu_2_O hetero-nanostructures. HRTEM images taken from central region (**c**) and tip region (**d**) of a single Ag-Cu_2_O nanoparticle.

To research the site-selective growth mechanism of AgNTs induced by light irradiation, Ag-Cu_2_O hetero-nanostructures are also synthesized following a photoinduced method. The plasmon wavelength of Ag-Cu_2_O hetero-nanostructures experiences a redshift and width broadening compared with the plasmon wavelength of AgNTs (Fig. 5). We have shown the high-resolution images of Ag-Cu_2_O hetero-nanostructures in Fig. 6. Evidently, the Cu_2_O nanoparticles are selectively grown at the tips of AgNTs.

High-resolution TEM images of the tip regions in the hybrid nanostructure are shown in Fig. 6. The lattice plane spacing of 0.3 nm in the tip region agrees well with the (110) lattice planes of Cu_2_O. The lattice plane spacing of 0.26 nm in the central region agrees well with the 1/3 (422) lattice planes of the face-centered cubic (fcc) Ag^58^. The inserted fast Fourier transform images also agree with the lattice planes for Cu_2_O and Ag, respectively.

Under the light irradiation, Ag-Cu_2_O nanocomposites with Cu_2_O nanoparticles on the tips are also obtained, which verifies the plasmon-assisted local field concentration for site-selective growth under the excitation of light. The thermal equilibration process takes places after irradiation, which results in high energy and activity at the tips of the nanoplates^56^. In this case, the reduced reaction of Cu stock tends to occur at the tips rather than on the surface or edge of AgNTs. Therefore, Cu_2_O nanoparticles prefer to deposit on the tips of AgNTs.

## Conclusions

In summary, we have shown that the site-selective growth of Ag leads to the tip-to-tip assembly of AgNTs in a large region by using a photoinduced method. The growth of Ag onto the tips of AgNTs under light irradiation is due to the concentrated local field induced by SPR. The AgNTs come to touch with each other at the tips and finally connect together into the branched chains. The redshifts of the plasmon band wavelength in the extinction spectra also agree well with the growth at the tips of AgNTs and the decrease of the gaps between the adjacent nanotriangles. We also synthesize the Ag-Cu_2_O nanocomposite under the condition of light irradiation and find that the Cu_2_O nanoparticles preferably grow on the tips of the AgNTs, which verifies that the local field concentration induced by surface plasmons with the excitation of light could be advantageous for the selective growth on special facets. The findings in this paper have potential applications in the bio-imaging, SERS and nanodevices.

## Methods

### Materials

Silver nitrate (AgNO_3_, ≥99.8%), cupric chloride (CuCl_2_∙2H_2_O, ≥99%) and ascorbic acid (AA, ≥99.7%) were purchased from Sinopharm Chemical Reagent Co. Ltd. (Shanghai, China). Sodium borohydride (NaBH_4_, ≥98%), (3-aminopropyl) trimethoxysilane (APTMS), polyvinylpyrrolidone (PVP) were stocked from Aldrich (America). Sodium citrate (Na_3_C_6_H_5_O_7_, ≥99%) was obtained from Shanghai Exhibition Cloud Chemical Co. Ltd. (Shanghai, China). All chemical materials were prepared without purification in our experiment.

### Preparation of AgNTs

AgNTs were prepared using a photochemical method which contains two steps^46^. First, in the preparation process of Ag seeds, 47.5 mL of ultrapure water was added into a round-bottomed bottle. Then, 500 uL of PVP (5 mg/mL), 1 mL of AgNO_3_ (5 mM) and 500 ul of Na_3_C_6_H_5_O_7_ (30 mM) were subsequently added into the bottle. Under magnetic stirring, 500 uL of fresh NaBH_4_ solution (50 mM) was introduced into the mixture. After 30-minute stirring, deep yellow color of Ag seeds solution was appear. Second, the bottle with Ag seeds solution was transferred and illuminated by a conventional 60 W table lamp. Finally, the reaction was stopped until the color of solution became blue. At last, the grown AgNTs were centrifuged twice at 10,000 rpm for 10 min. AgNTs were dispersed and store in water for further use.

### Growth and assembly of AgNTs on substrates under light irradiation

The prepared AgNTs were adsorbed on glass slides for aminosilanization following a method in ref. 43. In brief, the glass slides were cleaned and then deposed in ethanol solution with the presence of APTMS (1%, v/v). After 30 min, each glass slide was washed with ethanol and put in an incubator for 3 h. The reaction was kept at 120 °C. Then, the glass slides were transferred into AgNTs solution and kept undisturbed for 3 h. The glass slides were taken out for purification, following by put into the same AgNTs solution for the additional another 5 h adsorption of AgNTs. Each glass slide was irradiated with light from a Xe lamp with a bandpass filter (700 nm). The morphological changes of AgNTs at selected irradiation time are probed using SEM.

Similarly, the as-prepared AgNTs solution was dropped on the carbon-coated copper grids. Then the samples were also illuminated with the same light. The morphological changes of AgNTs at selected irradiation times are probed using TEM.

### Synthesis of Ag-Cu_2_O hetero-nanostructures under light irradiation

Ag-Cu_2_O hetero-nanostructures are also synthesized following a photochemical process. 5 mL of PVP (156 mg/mL) was injected into 5 mL of CuCl_2_ (0.32 mg/mL) aqueous solution. The mixture was allowed to keep in an oil bath for 5 min at 55 °C under slight stirring. 300 uL of mixture solution was put into a plastic tube. Then, 60 uL of NaOH (2 M) was injected into the tube, followed by 5 mL of redispersed AgNTs for 30 min. Subsequently, 600 uL of ascorbic acid (0.06 M) was also mixed into the tube. The solution was irradiated for 2 h by a 200 W high pressure mercury lamp. The products were centrifuged twice and dispersed in water.

### Characterization

The TEM images were taken on a JEOL 2010 HT transmission electron microscope operated at 200 kV. The SEM images were collected by using an FEG Sirion 200 scanning electron microscope with the accelerated voltage of 20.0 kV. Extinction spectra were monitored by a TU-1810 UV-Vis-NIR spectrophotometer (Varian, Cary 5000).

## Additional Information

**How to cite this article:** Xie, Y. *et al*. Plasmon-assisted site-selective growth of Ag nanotriangles and Ag-Cu_2_O hybrids. *Sci. Rep.* **7**, 44806; doi: 10.1038/srep44806 (2017).

**Publisher's note:** Springer Nature remains neutral with regard to jurisdictional claims in published maps and institutional affiliations.

## Supplementary Material

Supplementary Information
