# Supplementary material for: Plasmon-assisted site-selective growth of Ag nanotriangles and Ag-Cu2O hybrids
Source: Sci Rep. 2017 Mar 21;7:44806. doi: 10.1038/srep44806 (PMC5359615; doi:10.1038/srep44806)
Supplement: Supplementary Information [file srep44806-s1.pdf]

SUPPLEMENTARY INFORMATION

Plasmon-assisted site-selective growth of Ag nanotriangles and Ag-Cu_2O hybrids

Ying Xie[1], Liang Ma[1], Zi-Qiang Cheng[1], Da-Jie Yang[2], Li Zhou[1,*], Zhong-Hua Hao[1,*], and Qu-Quan Wang[1, 2,*]

[1]Key Laboratory of Artificial Micro- and Nano-structures of the Ministry of Education and School of Physics and Technology, Wuhan University, Wuhan 430072, P. R. China

[2]The Institute for Advanced Studies, Wuhan University, Wuhan 430072, P. R. China

[*]Correspondence should be addressed to Qu-Quan Wang, qqwang@whu.edu.cn; Li Zhou, zhouli@whu.edu.cn; Zhong-Hua Hao, zhhao@whu.edu.cn.

1. AgNTs prepared with varied reaction condition

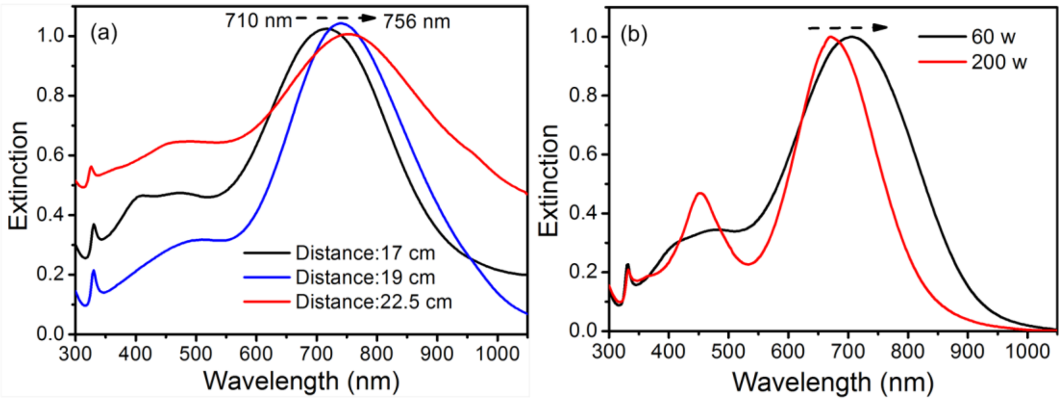

Supplementary Figure S1. UV-Vis-NIR extinction spectra of AgNTs prepared (a) with different distances from the light source to the reaction vessel, and (b) with different light powers.

Figure S1a shows the extinction spectra of the AgNTs prepared with different distances between the light source and the reaction vessel. As the distance between the light source and the reaction vessel is increased, the in-plane dipole resonance peak redshifts from 710 nm to 756 nm. Figure S1b shows the extinction spectra of the AgNTs prepared with different light powers. The in-plane dipole resonance peak is redshifted as the intensity of light is decreased. The redshift of the in-plane dipole resonance peak indicates the edge length of AgNTs is increases.

2. Growth of Ag in solution without light irradiation

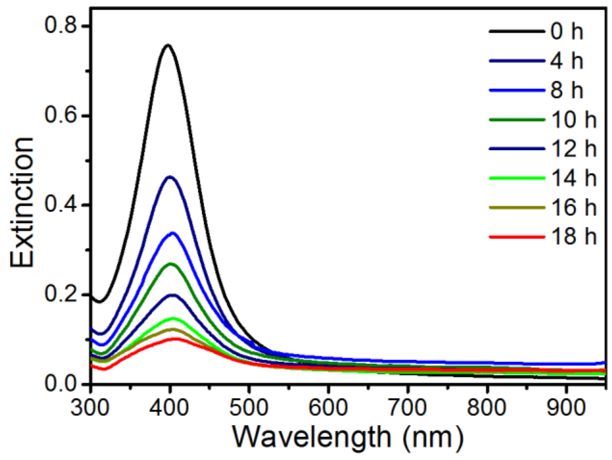

Supplementary Figure S2. Extinction spectra of silver seed solutions maintained at 30 °C for different time without light irradiation.

Figure S2 shows the extinction spectra of silver seed solutions measured at 30 °C without light irradiation. The peak wavelength is almost unchanged as reaction time increases. The results indicate that no AgNTs are formed without light irradiation.

3. Extinction spectra of AgNTs on glass slides with and without light irradiation

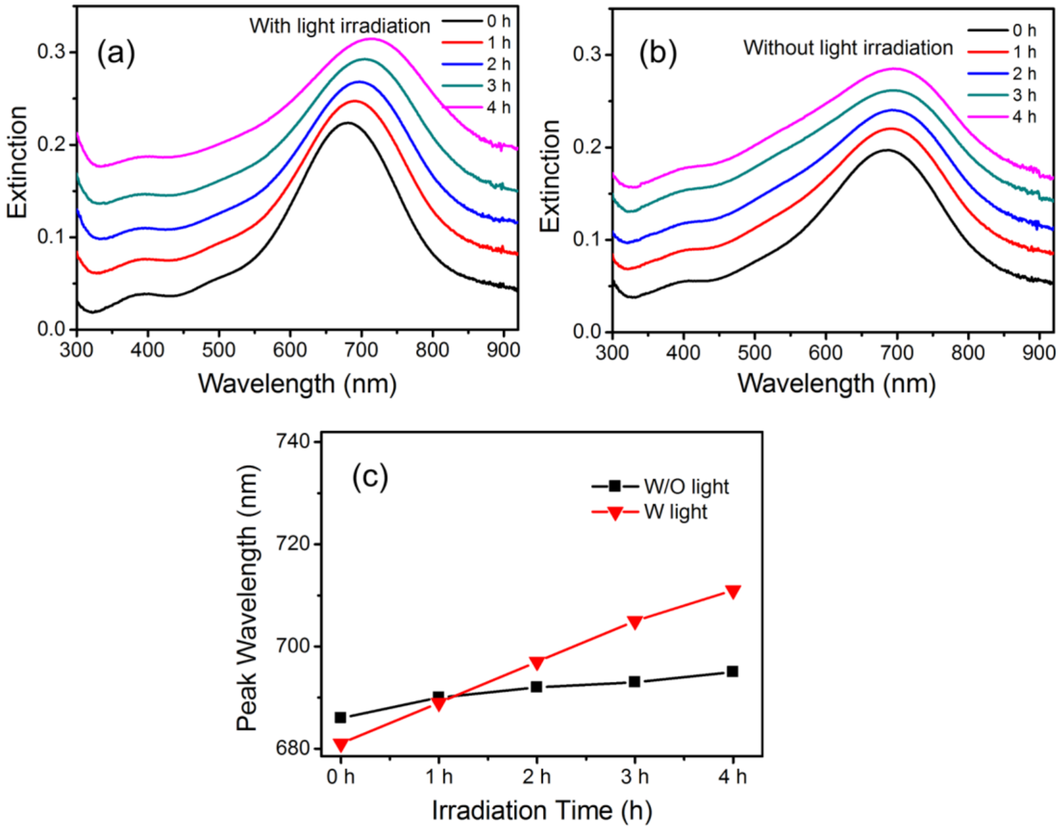

Supplementary Figure S3. Extinction spectra of AgNTs on glass slides with light irradiation (a), and without light irradiation (b). (c) Peak wavelength as a function of time.

The AgNTs absorbed on the glass slide are covered by a tin foil paper to remove photon influences. The plasmon band variations of samples on substrate with and without light irradiation are compared. As shown in Figure S3, the plasmon band redshifts 30 nm with light irradiation. The sample covered by tin foil paper only shows 9 nm shift. In our experiment, the AgNTs are bonded on the substrate and the aggregation cannot happen. The results reveal that the plasmon band redshift of AgNTs on substrate is mainly due to plasmon-assisted growth of Ag onto the AgNT tips.

4. SEM images of AgNTs on glass slides after light irradiation

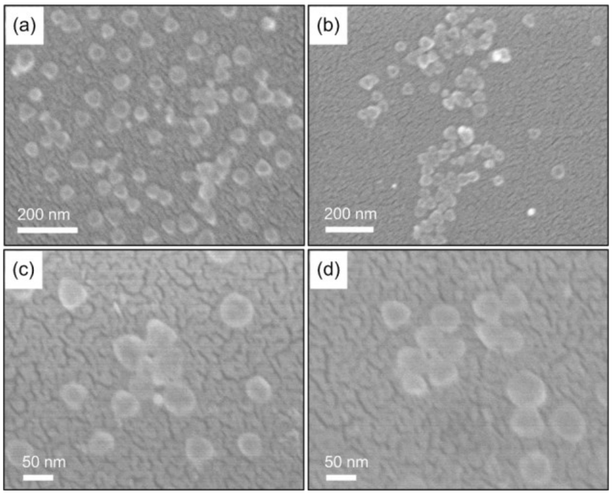

Supplementary Figure S4. (a)-(d) SEM images of AgNTs on a glass slide after light irradiation for 4 h on different regions.

Figure S4 shows the SEM images of AgNTs on a glass slide where more different morphologies can be seen after light irradiation. The overgrowth of Ag on the tips varies the shapes of AgNTs. With the increased illumination time, the site-specific growth of Ag leads to the assembly and "weld" of AgNTs in a large region.

5. TEM images of AgNTs on copper grids after light irradiation

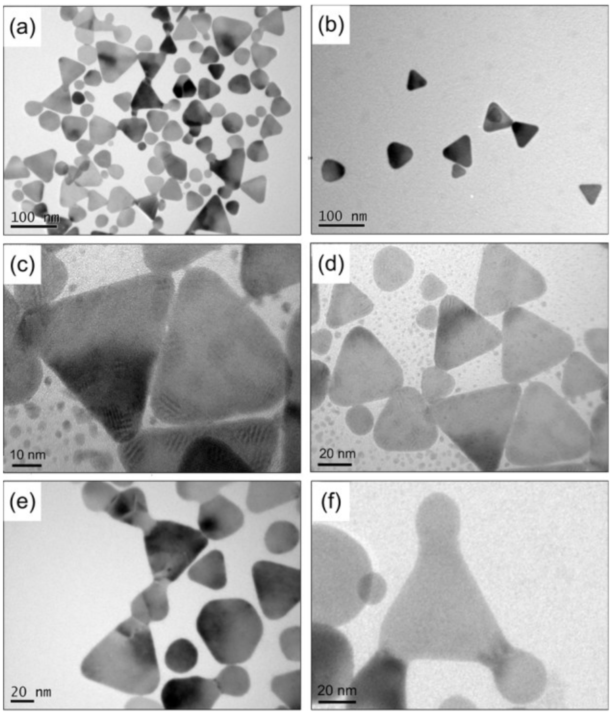

Supplementary Figure S5. (a)-(f) TEM images of AgNTs on copper grids after light irradiation.

Figure S5 shows the morphologies of AgNTs on copper grids after light irradiation. The overgrowth of Ag on the tips of AgNTs is clearly observed. Especially, the site-specific growth of Ag leads to the connection and assembly of the adjacent AgNTs, a “welding” phenomenon.
